# Clinical and microbiological characteristics of non-tuberculous mycobacteria diseases in Singapore with a focus on pulmonary disease, 2012-2016

**DOI:** 10.1186/s12879-019-3909-3

**Published:** 2019-05-17

**Authors:** Zoe Xiaozhu Zhang, Benjamin Pei Zhi Cherng, Li-Hwei Sng, Yen Ee Tan

**Affiliations:** 1 Department of Epidemiology, Medical Board, Singapore General Hospital, Singapore, Singapore; 2 Department of Clinical Epidemiology, Office of Clinical Epidemiology, Analytics, and kNowledge (OCEAN), Tan Tock Seng Hospital, Singapore, Singapore; 3 Department of Infectious Disease, Singapore General Hospital, Singapore, Singapore; 4 Department of Microbiology, Singapore General Hospital, Singapore, Singapore

**Keywords:** Non-tuberculous mycobacteria, *Mycobacterium abscessus*, Singapore

## Abstract

**Background:**

Information on non-tuberculosis mycobacterial (NTM) diseases remains limited in Singapore and other Southeast Asian countries. This study aimed to delineate epidemiological and clinical features of pulmonary NTM disease.

**Methods:**

A retrospective review was performed on all NTM isolates identified in Singapore General Hospital from 2012 to 2016 using the 2007 ATS/IDSA diagnostic criteria.

**Results:**

A total of 2026 NTM isolates from 852 patients were identified. *M. abscessus-chelonae group* (1010, 49.9%) was the most commonly isolated and implicated in pulmonary NTM disease. Pulmonary cases (352, 76%) had the highest prevalence among patients diagnosed with NTM diseases (465/852, 54.6%) with no gender difference. Male patients were older (68.5 years, *P* = 0.014) with a higher incidence of chronic obstructive pulmonary disease (COPD) (23.6%, *P* < 0.001) and recurrent cough with phlegm production (51.6%, *P* = 0.035). In contrast, more female patients had bronchiectasis (50%, *P* < 0.001) and haemoptysis (37.6%, *P* = 0.042). Age and COPD were associated with multiple NTM species isolation per patient.

**Conclusions:**

*M. abscessus-chelonae group* was the commonest NTM species isolated in Singapore. Pulmonary NTM infection has the highest frequency with male and female patients associated with a higher incidence of COPD and bronchiectasis respectively. Age and COPD were associated with multiple NTM species isolation per patient.

## Background

Non-tuberculous mycobacteria (NTM) has been increasingly implicated in a broad range of infectious diseases worldwide [1–4]. These environmental microbes are found primarily in water and soil. Possible route of transmission includes direct exposure to aerosolized water or soil containing NTM species [2–5]. The interaction between host immune system and the pathogenicity of the organisms plays a key role in disease susceptibility [6]. Well-defined host risk factors include advanced age, male gender, slender and older Caucasian women, immune defects, structural pulmonary diseases, alpha1-antitrypsin deficiency [7–11]. Urban living, especially in wet area with dense population, also increases the susceptibility to NTM infections [12, 13]. Other predisposing factors include low body-mass index, skeletal abnormalities and gastroesophageal reflux [11, 14].

Pulmonary, lymphadenitis, skin/soft tissue and dissemination are the four major clinical manifestations of NTM infections. Pulmonary NTM is the commonest clinical syndrome [15, 16] and it is associated with significant morbidity among older adults [7, 17]. Structural pulmonary diseases such as cystic fibrosis, chronic obstructive pulmonary disease (COPD) bronchiectasis, and tuberculosis (TB) predispose individuals to NTM lung infections. In the United States (US), an estimation of 86,000 NTM pulmonary cases were reported in 2010 with the prevalence of 8.6 per 100, 000 [18], and the number was growing by approximately 8% every year [14]. In Canada, the prevalence of NTM pulmonary disease was estimated to be 9.8 per 100,000 in 2010 [19]. Asians and Pacific Islanders appear to be more susceptible to NTM diseases [14]. Several studies have reported the increasing number of annual NTM isolates and the significant clinical relevance [20–22]. In Korea, the incidence of pulmonary NTM disease has doubled in 2015 compared to the number in 2009 [23, 24]. Japan revealed that NTM disease has reached 14.7 cases per 100, 000 people-years [25] . Pulmonary NTM disease has been described common in Taiwan, with the average incidence rate of 46.0 episodes per 100,000 hospital-based patient-years from 2010 to 2014 [26]. The epidemiology of NTM pulmonary disease differs in various geographical regions. The data remains limited in Southeast Asian countries including Singapore.

Singapore is a highly urbanized city-state with a tropical climate. It is the world’s third most densely populated country with a total population of 5.6 million. The percentage of the population aged ≥65 years is over 10% and is projected to grow to nearly 20% by 2030 [27]. In addition, a large proportion of residents aged over 50 years have a history of TB as TB was prevalent in Singapore until the 1970s [28]. Collectively, high population density, a wet tropical urban environment, and a large aging population with history of TB make the residents of Singapore particularly susceptible to NTM diseases. In this study, we evaluated the epidemiological and clinical characteristics of NTM diseases in Singapore, with an emphasis on pulmonary infection.

## Methods

### Study setting and study subjects

Singapore General Hospital (SGH) is a 1700-bedder tertiary hospital located within the Outram Campus in Singapore with 5 other National Specialty Centres, facilitating a comprehensive range of medical services for over 1 million patients annually. SGH CTBL is one of the two centralized laboratories accredited for mycobacterial testing in Singapore and is responsible for about 80% of the country’s mycobacterial culture testing volumes. All NTM isolates identified in SGH CTBL from 1st January 2012 to 31st December 2016 were included in the study. These were speciated by a combination of methods including high-performance liquid chromatography (HPLC; Beckman Instruments, USA), INNO-LiPA Mycobacteria v2 (Innogenetics, Belgium) and/or DNA probes (AccuProbes; Gen-Probe, USA) and correlated with the colonial morphology, pigmentation and growth rate. For cases with multiple isolates of one or more species, only the first isolate of the same species was included for analysis. *M. gordonae*, a well-known environmental contaminant, was excluded from the analysis [29–31]. Isolates were cultivated from various anatomical sites which included pulmonary source (sputum, bronchoalveolar lavage (BAL), lung biopsy, endotracheal tube aspirate (ETTA) and pleural fluid) and extrapulmonary sources such as skin and soft tissue, blood, lymph node and other sterile sites.

### Data collection

Medical records of included cases were reviewed from the electronic medical record system by trained research coordinators with nursing or medical background. The following information were collected: 1) Patients’ demographic information, 2) medical comorbidities, 3) clinical presentations, 4) radiology findings of nodular, cavitary opacities or bronchiectasis on chest X-ray (CXR); or bronchiectasis with multiple small nodules on high resolution computed tomography (HRCT) scan, and 5) microbiology results. In addition, history of tuberculosis, body-mass index (BMI) were also recorded.

NTM diseases were defined using the criteria proposed by 2007 American Thoracic Society and the infectious Diseases Society of America (ATS/IDSA) and were further categorized as pulmonary disease, skin/soft tissue disease, disseminated disease (blood/other sterile sites infection), lymphadenitis. NTM pulmonary disease was defined when patients had a combination of clinical, radiological, and microbiological features [32].

### Statistical analysis

Categorical variables were expressed as counts (percentage). Differences in frequencies were compared using a X^2^ test or Fisher’s exact test. Continuous variables were expressed as median with 25-75th interquartile range (IQR), and the difference was assessed using Mann-Whitney-U test. Univariate analysis was performed to assess the association between risk factors and multi-species infection, and the results were presented as odds ratios (ORs) with 95% confidence intervals (95% CIs). All the analyses were done using STATA, version 13 for Windows.

## Results

### Specimen source and NTM species distribution

A total of 2026 NTM isolates from 852 patients were identified during the 5-year study period. The specimen types and detected species were shown in Tables 1 and 2 respectively. Respiratory specimens were predominant (1777, 87.7%). The top four prevalent species were *M. abscessus-chelonae group* (1010, 49.9%), *M. fortuitum* group (345, 17%), *M. avium* complex (MAC) (309, 15.3%), and *M. kansasii* (233, 11.5%).

**Table 1:** Sources of the specimens (*N* = 2026)

Specimen source	Sub-total N (%)	Total N (%)
Pulmonary		
Sputum	1519 (75)	1777 (87.7%)
Broncho alveolar lavage	195 (9.6)	
Lung biopsy	39 (1.9)	
ETT aspirate	12 (0.6)	
Pleural fluid	12 (0.6)	
Extra-pulmonary		
Skin/soft tissue	96 (4.7)	249 (12.3%)
Blood/sterile sites	138 (6.8)	
Lymph node	15 (0.74)	

Note: the data was presented as number (%) otherwise specified

**Table 2:** NTM species distribution of all the investigated isolates (*N* = 2026)

Organism	No. of counts (%)
*M. abscessus-chelonae group*	1010 (49.9)
*M. fortuitum group*	345 (17.0)
*M. avium complex*	313 (15.4)
*M. kansasii*	233 (11.5)
*M. haemophilum*	41 (2.0)
*M. scrofulaceum*	22 (1.1)
*M. mucogenicum*	16 (0.8)
*M. lentiflavum*	14 (0.7)
*M. triplex*	10 (0.5)
*M. interjectum*	7 (0.3)
*M. simiae*	7 (0.3)
*M. marinum*	5 (0.2)
*M. terrae complex*	5 (0.2)
*M. szulgai*	4 (0.2)
*M. genavense*	2 (0.1)
*M. neoaurum*	2 (0.1)
*M. celatum*	1 (0.05)

Note: the data was presented as number (%) otherwise specified

### NTM diseases

**Fig. 1:**
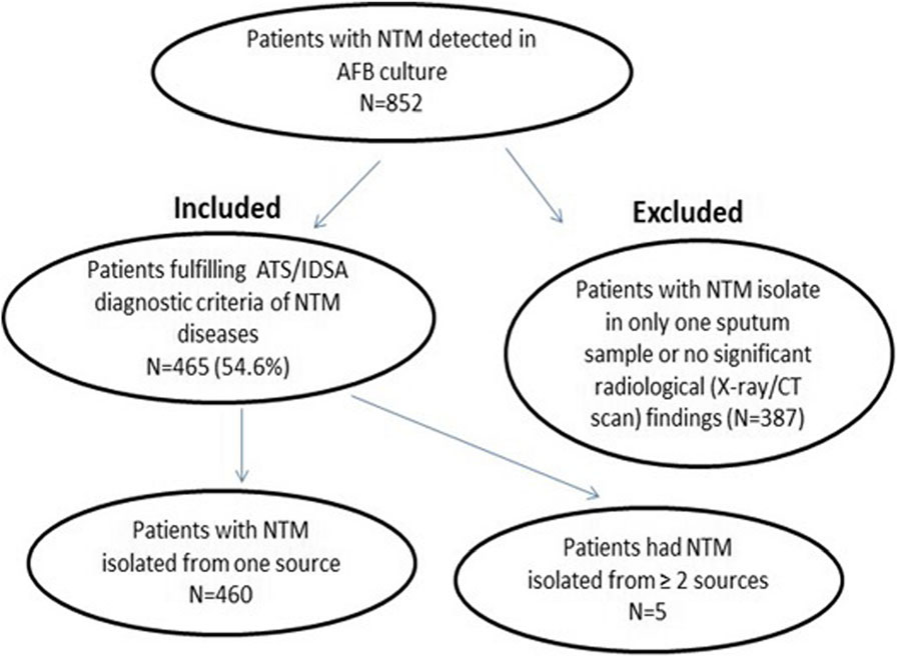
The study flowchart

**Fig. 2:**
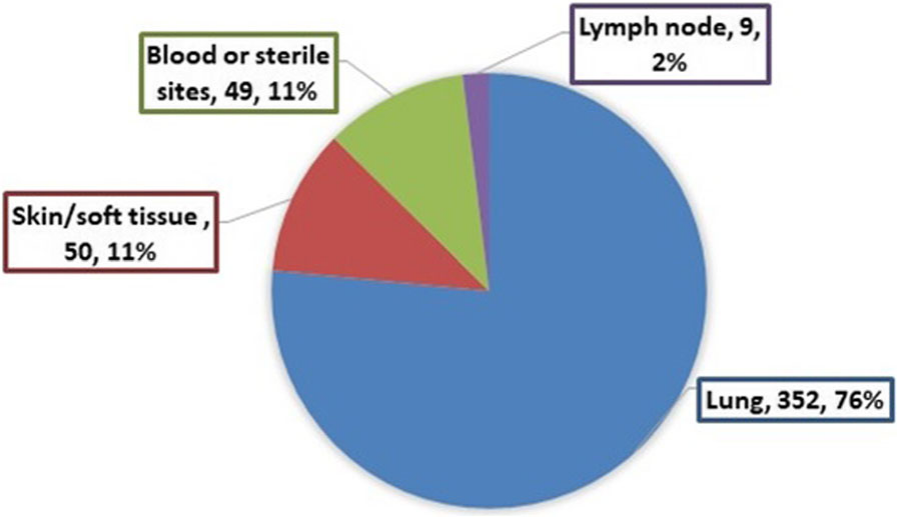
The distribution of NTM diseases among the study subjects

The study flowchart is shown in Fig. 1. Due to single sputum culture or the absence of characterized radiological changes as described in ATS/IDSA guideline, 387 patients with NTM isolate(s) from respiratory specimens (45.3%) were excluded from further analysis. Among the rest 465 patients (54.6%) who fulfilled ATS/IDSA diagnostic criteria, 460 patients had NTM isolated from one source, whereas 5 other patients had NTM isolated from ≥2 sources. The detailed disease distribution was illustrated in Fig. 2. Comorbidities of patients with NTM isolates from blood/other sterile sites mainly consisted of HIV/intro-venous drug abuse (11/49, 22.4%), malignancies (10/49, 20.4%), end stage renal failure (10/49, 20.4%), and ischemia heart disease /chronic heart failure (9/49, 18.4%). The species and isolates distribution of patients with NTM diseases were listed in Tables 3 and 4.

**Table 3:** NTM species distribution among the patients diagnosed with NTM diseases (*N* = 460)

Species	Pulmonary *N* = 352 (%)	Skin/soft tissue *N* = 50 (%)	Blood/ other sterile sites *N* = 49 (%)	Lymph node *N* = 9 (%)	Total *N* = 460 (%)
M. chelonae -M. abscessus group	197 (56)	25 (50)	16 (32.7)	6(66.7)	244 (53)
*M. avium* complex (MAC)	99 (28.1)	4 (8)	14 (28.6)	1 (11.1)	118 (25.7)
M. fortuitum species group	91 (25.9)	7 (14)	15 (30.6)	0	113 (24.6)
M. kansasii	71 (20.2)		1 (2.0)	1 (11.1)	73 (15.9)
M. scrofulaceum	8 (2.3)				8 (1.7)
M. mucogenicum	6 (1.7)		2 (4.1)		8 (1.7)
M. lentiflavum	3 (0.9)				3 (0.7)
M. szulgai	2 (0.6)				2 (0.4)
M. interjectum	1 (0.3)				1(0.2)
M. simiae	1 (0.3)	1(2.0)			2 (0.4)
M. triplex	1 (0.3)				1(0.2)
M. genavense				1 (11.1)	1(0.2)
M. haemophilum		10 (20)	2 (4.1)		12 (2.6)
M. marinum		3 (6.0)			3 (0.6)
M. neoaurum			2 (4.1)		2 (0.4)
M. terrae complex		1 (2.0)			1(0.2)

Note: the data was presented as number (%) otherwise specified

**Table 4:** Baseline characteristics of the patients with pulmonary NTM disease (*N* = 352)

Characteristics	No. of subjects (%)
Male, No. (%)	182 (51.7)
Median Age (IQR) (years)	67 (58–74)
Median BMI (IQR)	18.8 (16.2–21.5)
Ethnic groups	
Chinese	306 (86.9)
Malay	19 (5.4)
Indian	11 (3.1)
Others	16 (4.5)
Coexisting illness	
Transplant	7 (2.0)
HIV	13 (3.7)
Ischemia heart disease	42 (11.9)
Congestive heart failure	7 (2.0)
Malignant diseases	96 (27.3)
Renal disease	11 (3.1)
Liver disease	9 (2.6)
Diabetes mellitus	43 (12.2)
Cardiovascular disease	42 (11.9)
COPD	46 (13.1)
Bronchiectasis	128 (36.4)
Asthma history	14 (4.0)
TB history	97 (27.6)
Cerebrovascular disease	14 (4.0)
Clinical presentations	
Cough	261 (74.1)
Recurrent cough with phlegm production	160 (45.5)
Haemoptysis	114 (32.4)
Weight loss	28 (8.0)
Night sweat	7 (2.0)
Laboratory Investigations	28 (8.0)
AFB smear (+)	113 (32.1)
Blood culture (+)	3 (0.9)
Sputum culture (+)	30 (8.5)
Radiological findings	
Chest X-ray (*N* = 330)	
Fibrocavitary	25 (7.6)
Nodular bronchiectasis	144 (43.6)
CT scan (*N* = 260)	
Fibrocavitary	27 (10.4)
Nodular bronchiectasis	255 (98.1)

Note: the data was presented as number (%) otherwise specified;

*BMI* body mass index, *COPD* Chronic pulmonary obstructive disease, *TB* tuberculosis, *HIV* Human immunodeficiency virus

### NTM pulmonary diseases

#### Demographic information and clinical features

Of the 744 patients with NTM isolates from respiratory specimens, 352 patients (47.3%) met ATS/IDSA case criteria for pulmonary NTM disease. *M. abscessus*-*chelonae* group (197, 56%) was the predominately pathogenic species. The median age of the patients was 67 years (Table 4). Malignant diseases, COPD and bronchiectasis were the major comorbidities. Male patients were older, and more often had COPD; whereas female patients more often had bronchiectasis (Table 5).

**Table 5:** Comparison between male and female patients with NTM lung disease (*N* = 352)

Characteristics	Male (*N* = 182)	Female (*N* = 170)	*P* value
Median Age (25 th-75th IQR) (years)	68.5 (61–76)	66 (56–73)	0.014*
Median BMI (25th–75th IQR)	18.9 (16.2–22.3)	18.6 (16.1–21.3)	0.711
Coexisting illnesses No. (%)			
HIV	11 (6.0)	2 (1.2)	0.021*
Ischemia heart disease	34 (18.7)	8 (4.7)	< 0.001*
Neoplastic disease	48 (26.4)	48 (28.2)	0.7
Diabetes mellitus	29 (15.9)	14 (8.2)	0.028*
COPD	43 (23.6)	3 (1.8)	< 0.001*
Bronchiectasis	43 (23.6)	85 (50.0)	< 0.001*
TB history	64 (35.2)	33 (19.7)	0.001*
Clinical symptoms			
Cough	141 (77.5)	120 (70.6)	0.14
Recurrent cough with phlegm	94 (51.6)	66 (38.8)	0.035*
Haemoptysis	50 (27.5)	64 (37.6)	0.042*
Weight loss	38 (20.9)	33 (19.4)	0.73
Chest pain	17 (9.3)	11(6.5)	0.33
Investigations			
Median albumin (25th–75th IQR)	32 (27–37.2)	34 (28–40)	0.076

Note: the data was presented as number (%) otherwise specified. Univariate analysis was performed to assess the difference in observed variables between male and female patients with NTM diseases. Categorical variables were tested by Chi-square test or Fisher’s exact test if the frequencies were less than 5*. Continuous variables were examined using non-parametric Mann-Whitney test

*BMI* body mass index,*COPD* chronic pulmonary obstructive disease,*TB* tuberculosis,*HIV* human immunodeficiency virus

### Factors associated with multiple NTM species detection

Of the 352 patients with pulmonary NTM infection, 106 patients had ≥2 NTM species isolated either in the same setting (41/106) or at different time point (65/106). There were no species matching-pattern or sequential order observed. However, univariate analysis revealed that age ≥ 65 years and COPD were associated with multiple NTM species isolation with OR of 1.7 and 2.2 respectively (Table 6).

**Table 6:** Comparison of NTM lung disease characteristics between patients with 1 NTM species detected and patients with more than 1 NTM species detected

Characteristics	1 species (*N* = 246)	≥ 2 species (*N* = 106)	*P* value	OR (95% CI)
Male (%)	123 (50)	59 (55.7)	0.33	
Age ≥ 65 years	136 (55.3)	72 (67.9)	0.027*	1.7 (1.1–2.8)
Median BMI (25th–75th IQR)	19.1 (16.4–21.7)	18.1 (15.3–21.4)	0.14	
Coexisting illnesses No. (%)				
HIV	11 (4.5)	2 (1.9)	0.36	
IHD	28 (11.4)	14 (13.2)	0.63	
Neoplastic disease	73 (29.7)	23 (21.7)	0.12	
Diabetes mellitus	28 (11.4)	15 (14.2)	0.47	
COPD	25 (10.2)	21 (19.8)	0.013*	2.2 (1.2–4.1)
Bronchiectasis	87 (35.4)	41 (38.7)	0.55	
TB history	67 (27.2)	30 (28.3)	0.84	
Clinical symptoms No. (%)				
Cough	181 (73.6)	80 (75.5)	0.71	
Phlegm	110 (44.7)	51 (48.1)	0.56	
Haemoptysis	76 (30.9)	38 (35.8)	0.36	
Weight loss	50 (20.3)	21 (19.8)	0.91	
Chest pain	21 (8.6)	7 (6.6)	0.53	

Note: The data was presented as number (%) unless otherwise specified. Univariate analysis was performed to assess the difference in observed variables between male and female patients with NTM diseases

*BMI* body mass index,*COPD* chronic pulmonary obstructive disease,*TB* tuberculosis,*HIV* human immunodeficiency virus

## Discussion

In this study, we evaluated the clinical relevance of all NTM isolates cultured in CTBL in SGH from 2011 to 2015. We found that 54.6% of the patients with NTM isolates met ATS/IDSA diagnostic criteria of NTM diseases. Nearly half of the patients with pulmonary isolates may have active NTM lung infections, significantly higher than the rates reported in Hong Kong and Korea (47% vs. 20–25%) [20, 33]. This may suggest that pulmonary NTM isolates were more likely associated with pulmonary NTM infection rather than colonization among the patients in Singapore.

The distribution of NTM species varies geographically. *M. avium* complex (MAC) are the most commonly isolated and involved in pulmonary NTM infections in the US, Canada, Japan, Korea, Hong Kong and Taiwan [22, 32–40]. In contrast, our study showed that *M. abscessus*-*chelonae* group was the most prevalent and most implicated in pulmonary NTM disease among the patients in Singapore. Here we grouped *M. chelonae* and *M. abscessus* together as the two species could not be reliably differentiated by the line-probe assay (INNO-LiPA). However, an in-house review of isolates that were tested by both INNO-LiPA and HPLC revealed that all *M. abscessus*-*chelonae* group identified by INNO-LiPA were confirmed to be *M. abscessus* by HPLC method (Data not published). In view of this and the fact that *M.* c*helonae* rarely causes chronic lung disease [32], *M. abscessus* was likely to be the main pathogenic species in pulmonary NTM infections in our patient cohort. This observation was in line with another study in Singapore and a study conducted in southernmost region of Japan with subtropical climate [41, 42]. We postulated the higher prevalence of *M. abscessus* in our setting may be due to the tropical climate, densely populated urban living and history of TB infection. However, more studies are required to prove these hypotheses.

*M. abscessues* subsp*. abscessus* is often associated with poor clinical prognosis because of the possession of a functional *erm* [41] gene that confers macrolide resistance. *M. abscessus* subsp. *massiliense*, on the other hand, generally remains susceptible to macrolide due to the truncation of *erm* gene [43, 44]. Subspeciation of *M. abscessus* complex may provide important guidance in the clinical management of *M. abscessus* complex infection. Unfortunately, this data is not available during the study period as the test was not performed routinely*.* In addition, macrolide resistance is not routinely tested. If clinically indicated, the clinicians will order susceptibility testing for *M abscessus* and the microbroth dilution plate will be kept for a total of 14 days to detect inducible macrolide resistance. This is in accordance to CLSI (Clinical and Laboratory Standards Institute) M24-A2 [45].

In this study*, M. fortuitum species group* was detected in about 25% of the patients with pulmonary NTM diseases. The role of *M. fortuitum* group as the causative agent in infection can be controversial because it could be colonization or transient infection [46, 47]. However, some studies have reported that *M. fortuitum* is the pathogen implicated in pulmonary NTM infection and skin and bone/joint infection as well [32, 48–50]. Therefore, it should always be correlated with the clinical findings to determine its clinical significance and management. In addition, *M. kansasii is* a virulent but an uncommon species in Asia. However, it was surprisingly the fourth commonest NTM species implicated in pulmonary infection among the patients in Singapore [20, 22, 33]. Hence, it is important to understand the local epidemiology before applying guidelines elsewhere.

Structural lung diseases predispose people to pulmonary NTM infection [9, 14, 34, 51–56]. In this study, about 50% of patients with pulmonary NTM infection had COPD or bronchiectasis. Male patients had a higher incidence of COPD and more often presented with recurring cough and sputum production; in contrast, female patients had bronchiectasis and haemoptysis more frequently. These distinct features were consistent with the findings observed in other studies [34, 57, 58]. Pulmonary tuberculosis is another important pathophysiological process associated with severe pulmonary structural damage. A history of tuberculosis is a distinct characteristic among Asian patients with pulmonary NTM diseases [59]. In this study, one-third of the patients with pulmonary NTM infections had prior TB, which was not surprising as the incidence of TB in Singapore was about 307 cases per 100,000 population in 1960 [28]. By the way, the prevalence of pulmonary NTM disease was similar in male and female patients. Both male and female patients had slim figure with BMI close to the lower end of the normal range (18.9 kg/m^2^ and 18.6 kg/m^2^ respectively).

Isolation of multiple NTM species in the same setting or over a period of time is another feature of NTM lung infection and the underlying reason is unclear [60–62]. In this study, around 30% of the pulmonary NTM patients had ≥2 species identified. As clinical treatment data was not gathered in current study, we cannot tell if was related to clinical treatment as suggested by Lee et al. [63] However, we found that age ≥ 65 years and COPD were significantly associated with multispecies isolation with OR of 1.7 and 2.2 respectively. Chronic inflammation and remodelling of airways in COPD may play a role in the increased susceptibility to NTM infection.

This study has several limitations. Firstly, this is a retrospective study and the collected data may be incomplete. Secondly, the study was conducted in a single hospital not all adult tertiary-care hospitals in Singapore. Nonetheless, as patients from all over Singapore can seek care from any tertiary-care hospital in the country, any selection bias was likely to be minimal. Thirdly, there may be an underestimation in the prevalence of pulmonary NTM infections as approximately half of the cases with single NTM isolate from sputum or had no characteristic radiological findings were excluded from the analysis based on ATS/IDSA guideline.

## Conclusions

About half of the patients in Singapore with NTM isolates met ATS/IDAS criteria of NTM diseases with highest prevalence of pulmonary NTM disease. Of the 16 NTM species (group) detected in this study, *M. abscessus-chelonae group* was the most frequently isolated and most involved in pulmonary NTM diseases. The patients with pulmonary NTM disease were characterized with advanced age, having history of TB and other chronic illnesses such as malignant diseases and structural lung diseases. Male patients were more associated with COPD, whereas female patients more often had bronchiectasis. Increasing age and COPD were associated with multiple NTM species detection per patient.

## Abbreviations

AFB: Acid- Fast Bacilli
ATS/IDSA: American Thoracic Society/Infectious Disease Society of America
BAL: Bronchoalveolar lavage
BMI: Body-mass index
CI: Confidence interval
COPD: Chronic obstructive pulmonary disease
CTBL: Central Tuberculosis Laboratory
CXR: Chest X-ray
DM: Diabetes mellitus
ETTA: Endotracheal tube aspirate
HRCT: High resolution computed tomography
IHD: Ischemic heart disease
IQR: Interquartile range
MAC: *M. avium* complex
NTM: Non-tuberculous mycobacteria
OR: Odds ratio
SGH: Singapore General Hospital
TB: Tuberculosis
TNFα: Tumour necrosis factor alpha
